# The Impact of Ex-Post Legislative Evaluations in Healthcare: A Mixed Methods Realist Evaluation Study Protocol for Conducting Case Studies

**DOI:** 10.1177/16094069231184126

**Published:** 2023-06-29

**Authors:** Linda J. Knap, Johan Legemaate, Roland D. Friele

**Affiliations:** 1 Netherlands Institute for Health Services Research (NIVEL), the Netherlands; 2 Tranzo Scientific Center for Care and Wellbeing, Tilburg University, the Netherlands; 3 Law Centre for Health & Life, University of Amsterdam, the Netherlands

**Keywords:** realist evaluation, mixed methods, ex-post legislative evaluation, impact, the Netherlands, healthcare

## Abstract

**Background:**

Recent studies on the impact of ex-post legislative evaluations show that there are different types of impact and different factors that can influence it. These include the context of a legislative evaluation, research quality, and interactions between researchers and other actors within the evaluation process. However, thorough empirical research in this area is lacking. This warrants empirical research into the factors that influence the impact of ex-post legislative evaluations, so these insights can be used to increase the likelihood of ex-post legislative evaluations having an impact.

**Methods and analysis:**

In this protocol, we report on the realist evaluation methodology that will be used to evaluate the impact of three ex-post legislative evaluations in the Dutch healthcare sector. The mixed methods realist evaluation approach will facilitate this theory-driven, qualitative research. The study will consist of the following three steps: (1) Initial programme theory development, (2) theory validation, and (3) theory refinement. Knowledge from two scoping reviews conducted previously, and two subsequent expert meetings will form the basis for developing the initial programme theory. During this study, three case studies will be conducted, in which three individual ex-post legislative evaluations will be examined. Specificmethods for data collection will include: documentary review, observation, structured questionnaires and focus group discussions with purposefully identified key stakeholders. Using the framework approach, the data will be analysed thematically in a within-case analysis followed by a cross-case analysis.

**Discussion:**

This protocol provides insight into how the study will be conducted. As this study uses multiple qualitative researchmethods to answer one question, this protocol supports refining data collection procedures. Careful consideration of the approach beforehand can minimise pitfalls, reduce publication bias and improve reproducibility. The protocol therefore specifies how the research question will be answered in detail, and this provides solid guidance for the research process.

## Background

In this article, we report on a protocol for a realist evaluation study into the factors that may influence the impact of ex-post legislative evaluations (hereafter also referred to as ‘legislative evaluations’) in the Dutch healthcare sector. Legislation is an important and constantly evolving government instrument. It regulates healthcare, among other things, and directly affects the people involved, such as healthcare providers and patients. This justifies the need to check whether legislation actually does what it is supposed to after it has entered into force. To this end, ex-post legislative evaluations are conducted to examine the effectiveness of a law. They do so by ascertaining whether the law’s stated objectives have been achieved and what effects it has in practice. In order to improve the evidence base for healthcare legislation, the importance of conducting legislative evaluations is recognised globally. Consequently, legislative evaluations are being conducted to an increasing extent.

In the Netherlands, the evaluation of legislation is an increasing trend. In line with the broader development of legislative evaluations, the Netherlands has run the ZonMw programme for the evaluation of health laws and regulations since 1997. ZonMw is a Dutch funding organisation for innovation and research in healthcare. The programme is designed to contribute to the quality of health law legislation. Legislative evaluations are initiated at the request of the Ministry of Health, Welfare and Sport. They are carried out by independent, multidisciplinary research groups, selected for each study by the ZonMw regulatory evaluation committee. For each evaluation, ZonMw appoints an advisory committee with several experts from the field. This committee oversees the research process and acts as a sounding board. After the completion of the evaluation study, the evaluation report is presented to the ZonMw regulatory evaluation committee and the Ministry of Health, Welfare and Sport.

With nearly a quarter of a century’s experience in conducting legislative evaluations using this programme, an important question has arisen: what is the actual impact of these evaluations, and what mechanisms are in place to support this impact? Within the ZonMw programme, numerous evaluations have been conducted with the aim of implementing the gained insights. However, it remains to be seen whether and how this implementation takes place effectively.

Prior to writing this protocol, an extensive literature research was conducted to provide insight into the existing knowledge on the topic. Two scoping reviews (Knap et al., 2023b, 2023a) described what is currently known in the existing literature about the types of impact of ex-post legislative evaluations and about the factors that may influence them. It should be noted that these scoping reviews were based on a broad-based literature review, without limitation to a specific country or jurisdiction. The first scoping review showed that legislative evaluations fall within three domains: policy, politics and society (Knap et al., 2023a). Although society is directly affected by the presence of legislation, the impact of legislative evaluations seems to be particularly present in the domain of policy and politics. The findings of the second scoping review clarified the factors that influence the impact of legislative evaluations, namely context, research quality and interaction (Knap et al., 2023b). However, in contrast to extensive empirical research, more than a third of the data in both scoping reviews consisted of expert opinions. This justifies the need for empirical research into the factors that influence the impact of legislative evaluations. Since the evaluations carried out within the ZonMw programme potentially contain a great deal of useful information on this, it provides an excellent opportunity to conduct empirical research into evaluations of health legislation. The outcome of the second scoping review can thus be assessed in the context of a defined programme.

Given the factors mentioned above, the research question in this study will be: *What factors influence the impact of expost legislative evaluations in the Dutch healthcare sector?*

The methods section describes a theory-based protocol to conduct this empirical research. The study that will be conducted based on this protocol aims to better understand the mechanisms that provide insight into influenceable factors that can contribute to increasing the likelihood of legislative evaluations having an impact. Since legislative evaluations are conducted worldwide, this is an important contribution to the literature on the impact of legislative evaluations. By describing the research design in this protocol, we are compelled to thoroughly think about the rationale, approach and purpose of this study. Moreover, this protocol reduces publication bias and improves reproducibility.

The specific objectives of the study that follows based on this protocol are as follows:

1. To develop a literature-based and empirically validated theoretical framework to maximise the impact of expost legislative evaluations.
2. To develop an in-depth understanding of the influenceable factors that support the impact of Dutch healthcare ex-post legislative evaluations.
3. To identify, assess and compare the outcomes of different case studies in which the impact of Dutch healthcare ex-post legislative evaluations is studied.

## Methods and Analysis

A realist evaluation design is well suited to assessing how interventions work in complex situations because it allows the evaluator to deconstruct the causal web of conditions underlying them (Pawson & Tilley, 1997). These interventions are particularly useful for evaluating programmes that produce mixed outcomes, such as the ZonMw programme, to better understand how and why differential outcomes occur.

### Realist Evaluation

This study is designed as a mixed methods process and will be conducted on the basis of the Realist Evaluation (RE) method (Shamseer et al., 2016) developed by Pawson and Tilley (Pawson & Tilley, 1997). This method assumes that the same intervention will not work everywhere and for everyone. As opposed to the question of whether it works, this theory focuses on what works in what circumstances and for whom. The complete RE question is: “What works, for whom, in what respects, to what extent, in what contexts, and how?” In short, the key questions in RE are about causality and attribution. To answer these questions, realist evaluators aim to identify the underlying generative mechanisms that explain how the outcomes were caused and the influence of context.

The RE method consist of three key concepts: context, mechanisms and outcomes. Initially, a Context-Mechanism-Outcome (CMO) hypothesis about which mechanisms are likely to work in different contexts and which outcomes will be observed is developed. The context determines whether mechanisms work during a programme and may vary depending on various circumstances (e.g., social and political). Mechanisms intermediate between the concrete components of the interventions and the outcomes. They need the right context to work; any changes in the system can affect the causal process. The outcomes of a programme can be intended or unintended and can be short, medium and long-term. There can also be multiple outcomes of varying importance for different stakeholders. Both context and mechanisms must be systematically researched alongside interventions and outcomes.

The use of the RE method fits well with earlier research on the impact of ex-post legislative evaluations because it reflects the elements of context, mechanism and outcome mentioned above. The previously conducted scoping reviews show that context matters. In addition, there can be different types of impact (outcomes) and there are several factors (mechanisms) that can influence the impact of expost legislative evaluations. The RE method enables us to look deeper into the factors that can be influenced regarding the impact of legislative evaluations.

### Study Design and Setting

The 6-month study (April – September 2023) will be carried out in the Netherlands, building upon the 25 years of experience with legislative evaluations in the Dutch healthcare sector. Since realistic evaluation is method-neutral (Pawson & Tilley, 1997) and does not force the use of certain methods, in this study, a mixed methods approach is chosen for three case studies. Each case study examines one ex-post legislative evaluation from the ZonMw programme in detail. In this way, the case studies will focus on the Dutch situation, specifically within the Dutch healthcare sector.

## Methods of Data Collection

### Phase 1: Initial Programme Theory Development

The first phase is almost completed and consists of two parts: two scoping reviews and two expert meetings. Based on these inputs, an initial programme theory (IPT) will be developed that connects the context-mechanism-outcome (CMO) configurations. The IPT outlines how mechanisms work in a specific context to achieve certain outcomes, which fits well with this study’s aims.

The first scoping review showed that different types of impact of ex-post legislative evaluations could be distinguished in the literature; these can be divided into seven categories (see Table 1).

These types of impact can be connected to varying degrees and relate to different parties: the legislative community, policymakers and broader society. The second scoping review examined factors that can be influenced during the evaluation process. The results from this study showed various factors that can influence the impact of legislative evaluations (Knap et al., 2023b). These factors were divided into three categories: context, research quality and interaction (see Table 2).

The authors cited in this scoping review specifically mention the context in which an evaluation of legislation takes place. Contextual factors affect the evaluation process, but they are fixed and cannot be influenced by researchers. Factors that can be influenced by researchers are described and divided according to research quality (in a broad sense) and in terms of the interactions between researchers and stakeholders (Knap et al., 2023b). The influencing factors on the researchers’ side are the focus of this study. To test and further specify the findings in the literature, two expert sessions were held in which the recognition or absence of factors was discussed. These sessions also highlighted interest in research quality, with specific reference to research independence and how this relates to the impact of evaluation results and interactions between the researchers and stakeholders throughout the evaluation process. During these expert sessions, it was concluded that research quality and independence can be at odds with interactions. Both research quality and interactions were seen as modifiable factors. Therefore, these two factors were included as separate mechanisms in two CMO configurations.

Since the subject of this study is the evaluation process as a whole, the process from the creation of the evaluation proposal (initiation phase) to the dissemination of the results (implementation phase) will be included. The assumption in this IPT is that devoting attention to interaction and research quality during the evaluation process affects the impact of ex-post legislative evaluations. Two CMO configurations were prepared to test this IPT (see Table 3).

The IPT will be refined using these two CMO configurations. With regard to both CMO configurations, both the evaluation initiation phase and the actual implementation phase will be examined. The specific methods to examine these two phases within both CMO configurations are described in the section ‘*Research methods and respondents*’.

### Phase 2: Data Collection and Theory Validation

The initial IPT will be continuously validated and refined during data collection and analysis in Phase 2, which is planned to start in April 2023 and will last approximately six months. In this phase, three case studies will be carried out to understand and validate the above illustrated IPT that links context, mechanisms and outcomes of the impact of legislative evaluations. In each case study, an individual expost legislative evaluation from the ZonMw programme will be investigated. The three evaluations were chosen on the basis of time (not having been evaluated too recently or too long ago), diversity in whether or not the subject matter was ethical in nature, and the substance of the evaluation. Based on these considerations, the following ex-post legislative evaluations from the ZonMw programme will be examined:

- First evaluation of the Youth Act (January 2018)
- First evaluation of the Healthcare, Quality, Complaints and Disputes Act (February 2021)
- Third evaluation of the Embryo Act (March 2021)

In this phase, specific objectives were set to assess the CMO configurations:

1. Examine different types of impact of legislative evaluations, derived from a scoping review, in practice.
2. Identify the link between research quality and the impact of legislative evaluations.
3. Identify the link between interaction and the impact of legislative evaluations.
4. Examine other factors influencing the impact of legislative evaluations, derived from a scoping review, in practice.

## Research Methods and Respondents

To achieve these objectives, several qualitative research methods will be used. As mentioned earlier, this study will be conducted using two CMO configurations. The first CMO configuration is based on interaction (see Table 3). In this context, ‘interaction’ refers to productive interactions and mutual influence. It centres on interactions that lead to expectations among stakeholders and agenda-setting among researchers as an outcome of the actual interaction. The second CMO configuration is based on research quality (see Table 3). In this context, ‘research quality’ concerns the composition of the research group, the combination of legal and empirical research methods, the presence of different perspectives, and the recommendations made in the final research report. For both CMOs the whole evaluation process will be examined: from the initiation phase to the implementation phase.

### Document Review and Observation

First, an objective document review will be made of key documents resulting from the evaluation process. For the initiation phase, these documents include the programme text, accepted project proposal and feedback from an advisory committee. For the implementation phase, the products delivered by the research group will be examined, such as the final research report.

### Structured Questionnaires and Focus Groups

After the document review and observation, respondents will be asked to examine the CMOs subjectively. As noted earlier, different categories of actors could be derived from the first scoping review describing the different impact areas Knap et al., 2023a. These different groups of actors include both providers of legislative evaluations (i.e., researchers) as well as the users of the evaluation results (policymakers, politicians, legal community and society). In addition, there is the specific situation in the Netherlands that ex-post legislative evaluations are commissioned by an external party: ZonMw. This is a funding organisation of innovation and research in healthcare, such as the healthcare legislative evaluations, for which they have run the Evaluation Legislation and Regulation programme since 1997. The regulatory evaluation committee is responsible for implementing the programme as well as formulating the content of the evaluation assignments and selecting a multidisciplinary, independent research group for each legislative evaluation. ZonMw also appoints an advisory committee for each legislative evaluation that guides the evaluation process and acts as a sounding board. These advisory committees always include members of the regulatory evaluation committee of ZonMw. Owing to this special role in the evaluation process, members of the regulatory evaluation committee and the advisory committees will also be involved in this study.

A description of each group is provided in Table 4. For each individual case study, the specific individuals and organisations that belong to the seven groups identified in Table 4 will be indicated. This information will help to provide a clear understanding of the different perspectives and experiences involved in the evaluation process. In some cases, however, there may be overlap. For example, policymakers are considered users. However, in some cases, they can also be classified as commissioners. After Phase 1, a detailed list of respondents will be drawn up without divisions in terms of age or gender. Representatives from each of these seven different categories of actors will be included if they played an active role in the design or implementation of the evaluation, or if they belong to the groups of users of the law targeted by the evaluation.

Structured questionnaires that are partly open ended have been chosen because the questions are concrete and defined based on a fixed template, which specifies the exact wording and order of the questions (see Table 5 for the topics), and because this ensures that a larger population can be reached. In all three case studies, one questionnaire will be sent to each group of actors with a maximum of 100 people per group. This number is sufficient to capture key perspectives and achieve data saturation. The questionnaires align with the study’s four objectives (see Table 5). With regard to the distinction between the questions for the providers and the users, two different versions of the questionnaires will be developed. This approach ensures that the specific perspectives of both groups are adequately addressed.

### Theory Validation

In addition to data collection using structured questionnaires, a focus group discussion with key stakeholders will be held for each case study to validate the theory. During these focus groups, we will delve deeper into the possible factors and the context that influenced the impact of the evaluation in question with a number of key players, to ensure that our interpretations and conclusions are consistent with respondents’ views and experiences. Focus group discussions were chosen to uncover factors influencing opinions, behaviour or motivation (Krueger & Casey, 2015) and to compare the perspectives of different groups of actors in the three case studies.

The aim is to represent different groups of actors, but all actors’ experiences relate to the same case, so experiences can be exchanged. To enable all respondents to share insights and observations, the focus groups will consist of a maximum of 12 people (two from each group of actors) (Krueger & Casey, 2015). Group intelligence and deliberation will give us a more thorough understanding of these groups’ perceptions and reasoning (Manzano, 2022). There is no agreement in the literature on the optimal number of focus groups (Guest et al., 2017), the content and people spoken to is considered more important than the number of focus groups (Manzano, 2022); accordingly, three focus groups should be sufficient for this study. In this way, one overarching focus group is held for each case study (see Table 4). Since ZonMw’s regulatory evaluation committee largely consists of the same people for the three case studies, a separate focus group will be organised with them to discuss all three cases.

### Recruitment Strategies and Data Ethics

The questionnaires will be sent digitally, so respondents’ email addresses will first have to be collected. Some of these email addresses are publicly available, and for the non-public email addresses, we will approach our contacts at ZonMw and the Ministry of Health, Welfare and Sport. Respondents will be sent a single invitation to participate in the questionnaire. Should they fail to respond, they may receive up to two subsequent reminders. Completion of the questionnaire is voluntary and will not be financially compensated. The accompanying text informs the respondents about the purpose of the survey, the duration, the use of the data and the retention period. They will be asked to agree to these conditions prior to the questionnaire. Focus group participants will be invited to participate in a separate email.

The data received will be entered directly into a secured database, from which analyses can be carried out. The focus group discussions will be held online and, after informed consent is given, audio-recorded and transcribed. The audio file will be destroyed after transcription. As shown by a comparative analysis study, the content of the data generated from both online and in-person focus group discussions is remarkably similar (Woodyatt et al., 2016). As the focus groups will be held in Dutch, the excerpts used for the report will be translated into English.

As this study will involve human participants, ethical approval has been sought and received from the Ethics Review Board Tilburg School of Social and Behavioral Sciences [TSB_RP998] for this phase of the study.

### Phase 3: Data Analysis and Theory Refinement

**Figure 1:**
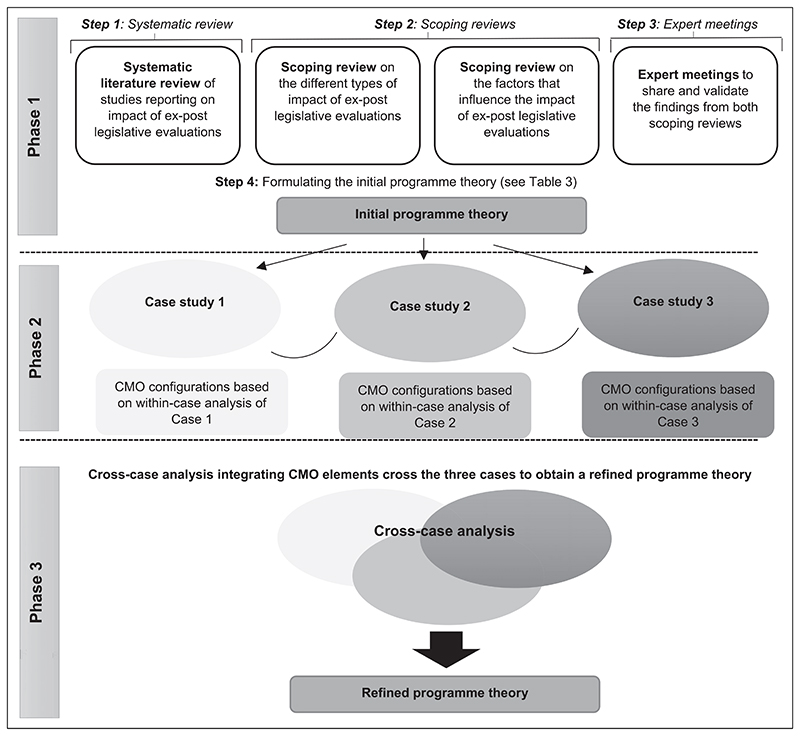
Schematic overview of research phases in realist evaluation (Mukumbang et al., 2018).

During this phase, the IPT will be refined based on an empirically tested CMO configuration. The research phases described above are represented schematically in Figure 1.

### Data Analysis

As the structured questionnaires and focus group discussions contain both closed-ended and open-ended questions, a combination of descriptive statistics, graphs and some nonparametric inferential statistics will be used for the data analysis. During the data analysis, the IPT will be leading. This means the questionnaire and focus group data will be analysed in light of the IPT. Based on the data analysis, it will be investigated whether there are additions to the IPT and whether they are widely supported.

### Within-Case Analysis

The data will be analysed thematically using the framework approach. This approach is suitable for studies using different qualitative approaches (Hackett & Strickland, 2019), such as the questionnaires and focus group discussions in this study. The framework analysis consists of five stages: (1) familiarisation, (2) identifying themes, (3) indexing, (4) charting and summarising, and (5) interpretation/mapping (Hackett & Strickland, 2019). First, the data will be analysed for each actor for the different topics and provided with a narrative for the fragments that are found to be related to each topic. The narratives will be summarised and inserted into the corresponding cell in a matrix (see Table 6). Subsequently, the information per topic can be compared for each actor involved. This allows the researchers to delve deep into the data of a single case and assess the different perspectives of different actors on each topic (see Table 6). Due to the exceptional role of the ZonMw regulatory evaluation committee both before and during the evaluation process, this data will be analysed separately.

### Cross-Case Analysis

After the within-case analysis, a cross-case analysis will be conducted comparing the different case studies. In this way, similarities and differences of perspectives between the three case studies regarding the topics can be identified (see Table 7). The interpretation of the research findings will consider whether the perspectives are consistent, partially consistent or inconsistent. It will also be examined whether new topics have emerged that are or are not widely supported.

Following the cross-case analysis, interpretations and summary findings of the analysis will be shared with all key stakeholders during a focus group discussion. This is discussed above as part of Phase 3. The CMO configurations will be refined based on both the within-case analysis and the cross-case analysis.

## Discussion

With this study, we aim to provide an in-depth understanding of the impact of ex-post legislative evaluations of Dutch health law, and specifically of the factors that may influence this impact. To this end, this study uses RE as the overarching conceptual framework to examine the actual impact of Dutch ex-post legislative evaluations in the healthcare sector. RE guides the development, validation and refinement of theories through analysis of the interplay between context, mechanisms and outcomes. In this way, the study sheds light on how the context of legislative evaluation implementation (e.g., evaluation initiation, function and political or social sphere) influences intervention mechanisms (e.g., research quality and interaction between researchers and stakeholders) to produce both intended and unintended outcomes. Since contextual factors are fixed and cannot be influenced, in this study, the RE method will be used to examine researcher-influenced factors related to the impact of ex-post legislative evaluations. The existing literature suggests that there are two influential factors: the quality of the research, in a broad sense, and the interaction between the parties involved in the research. The study described in this protocol will provide insight into the presence of these factors and the extent to which they can be influenced and, subsequently, if they affect the impact of ex-post legislative evaluations. Whether there are other factors that can significantly influence the impact of legislative evaluations will also be identified. The aim is to empirically validate and refine the factors that researchers can influence regarding the impact of expost legislative evaluations. In this way, the probability of evaluation research having an impact may be increased. As ex-post legislative evaluations are carried out worldwide, this could be a major contribution to the existing evaluation literature.

Gaps in the literature on the impact of legislative evaluations combined with practical issues raised by researchers and funders of evaluation studies provide a clear research focus. The current literature does not yet provide a solid basis for mapping the impact of legislative evaluations. The aim of this study, therefore, is to reflect on the basic theory (IPT), which is based on two scoping reviews and two expert meetings. This protocol transparently provides insight into how the study will be conducted. As this study uses multiple qualitative research methods (such as document review, structured questionnaires and focus group discussions) to answer a single question, this protocol provides support in refining data collection procedures. By carefully considering the approach beforehand, pitfalls can be minimised. The protocol therefore specifies in detail how the research question will be answered. This provides solid guidance during the research process. In addition, this protocol offers the possibility of replicating the study in other jurisdictions. The results of this study may also be of great interest to those involved in legislative evaluation in other countries since legislative evaluations are conducted worldwide.

While RE is a valid evaluative way of looking at the context, underlying mechanisms and outcomes of a complex intervention, there are also potential limitations. First, conducting the study in only one country and specific jurisdiction, in this case Dutch health law, may affect the generalisability of the study findings. However, RE applies the idea of generative causality, meaning that mechanisms only work if the context is conducive. It can identify the circumstances under which the intervention does or does not work, and how this happens. This allows policymakers to assess whether interventions that have proved successful in one setting can also be successful in another, and it helps them to adapt interventions to specific contexts. Second, RE implies continuous assessment of where to focus. Consequently, only a few ‘black boxes’ (mechanisms) can be unravelled. Concentrating on mechanisms may lead to bias but on the other hand, the focus is based on prior research. Moreover, it is impossible to unravel all mechanisms.

## List of abbreviations

CMO: Context-Mechanism-Outcome
IPT: Initial Programme Theory
Legislative evaluations: Ex-post legislative evaluations
RE: Realist Evaluation

## Figures and Tables

**Table 1:** Types of Impact of Ex-post Legislative Evaluations (Knap et al., 2023b).

Types of impact of ex-post legislative evaluations	
1	Knowledge and understanding
2	Confirmation of well-functioning legislation
3	Legislative revision
4	Influence on the legislative process
5	Influence on the policy process
6	Influence on the political sphere
7	Influence on society

**Table 2:** Factors that influence the impact of ex-post legislative evaluations (Knap et al., 2023b).

Factors that influence the impact of ex-post legislative evaluations	
1	Context
	Initiation and function of evaluation
	Openness to evaluation results
	Political and societal influence
2	Research quality
	Composition and independence of the research group
	Methods used
	Quality and content of evaluation report
3	Interaction
	Interaction between researchers and stakeholders
	Presentation and availability of research results
	Timing

**Table 3:** Initial programme theory.

Initial programme theory (IPT)				
Devoting attention to interaction and research quality during the evaluation process affects the impact of an ex-post legislative evaluation				
Context	+	Mechanism	=	Outcome
C_1_ – Evaluation initiation and function, characteristics of the law and legislative process, and the political and societal influence		M_1_ – Paying attention to and implementing *interaction* between researchers and stakeholders during the evaluation process		O_1_ – Impact of ex-post legislative evaluations (in the legislative community, policy area and broader society)
C_2_ – Evaluation initiation and function, characteristics of the law and legislative process, and the political and societal influence		M_2_ – Paying attention to and implementing *research quality* during the evaluation process		O_2_ – Impact of ex-post legislative evaluations (in the legislative community, policy area and broader society)

**Table 4:** Categories of Respondents Included.

Categories of respondents		
Group of actors	Description	Methodology
Providers		
Researchers	Individuals who are part of the research group conducting the legislative evaluation	Structured questionnaire and focus group discussion
Users		
Policymakers	Commissioning parties/policy officers at the ministry of health, welfare and sport	Structured questionnaire and focus group discussion
Politicians	Members of parliament, such as ministers and members of a political party who have the evaluation topic in their portfolio	Structured questionnaire and focus group discussion
Legal community	Lawyers, healthcare legal counsels, jurists and academics in the field of (health) law who are not involved in the legislative evaluation	Structured questionnaire and focus group discussion
Society	People in society who are subjects of the legislative evaluation, such as healthcare providers, patients and umbrella organisations within the healthcare sector	Structured questionnaire and focus group discussion
ZonMw advisory committees	ZonMw appoints an advisory committee for each legislative evaluation, which guides the evaluation process and acts as a sounding board	Structured questionnaire and focus group discussion
ZonMw regulatory evaluation committee	This committee consists of professionals whose activities relate in some way to healthcare legislation. These may include academics, jurists, policy officers and healthcare professionals	Focus group discussion

**Table 5:** Topics for Structured Questionnaires and Focus Group Discussions.

Topics for structured questionnaires and focus group discussions		
No.	Question topics	Relevant objective
1. Impact		
1.1	Types of observed impact	1
1.2	What respondents themselves did with the results and recommendations of the legislative evaluation	1
2. Factors		
2.1	Factors influencing the impact of legislative evaluations	4
2.2	Identifying reasons why respondents did or did not act on the results and recommendations of the legislative evaluation	4
2.3	Assessment of context, quality and interaction during and after the legislative evaluation	2–3

**Table 6:** Within-case analysis matrix.

	**Case study 1 :** First evaluation of the Youth Act					**Case study 2:** First evaluation of the Healthcare Quality, Complaints and Disputes Act					**Case study 3:** Third evaluation of the Embryo Act				
	Impact	Context	Research quality	Interaction	Other factors	Impact	Context	Research quality	Interaction	Other factors	Impact	Context	Research quality	Interaction	Other factors
**Researchers**															
**Policymakers**															
**Politicians**															
**Legal community**															
**Society**															
**ZonMw advisory committee**															

**Table 7:** Cross case analysis matrix.

	Cross-case analysis of all three case studies				
	Impact	Context	Research quality	Interaction	Other factors
**Researchers**					
**Policy makers**					
**Politicane**					
**Legal community**					
**Society**					
**ZonMw advisory committee**					

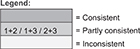

## Strengths and Limitations of this Study

- This is the first study that will provide insight into the influenceable factors that researchers can use to increase the likelihood of ex-post legislative evaluations having an impact in the Dutch healthcare sector.
- This paper should be relevant to researchers interested in adapting and applying a realist evaluation method to assessing complex interventions, such as ex-post legislative evaluations.
- Conducting the study in only one country and specific jurisdiction, in this case Dutch health law, may affect the generalisability of the study findings. However, the realist evaluation applies the idea of generative causality. This means mechanisms only work if the context is conducive. It can identify the circumstances under which an intervention does or does not work, and how this happens. This allows stakeholders to assess whether interventions that have proved successful in one setting can also be successful in another. It also helps to adapt interventions to specific contexts.
- Realist evaluation implies continuous assessment of where to focus. Consequently, only a few ‘black boxes’ (mechanisms) can be unravelled. Concentrating on mechanisms may lead to bias but, on the other hand, the focus is based on prior research. Moreover, it is impossible to unravel every mechanism.
